# Memory T Cell Migration

**DOI:** 10.3389/fimmu.2015.00504

**Published:** 2015-10-02

**Authors:** Qianqian Zhang, Fadi G. Lakkis

**Affiliations:** ^1^Tsinghua University School of Medicine, Beijing, China; ^2^University of Pittsburgh School of Medicine, Pittsburgh, PA, USA

**Keywords:** memory T cell subsets, lymphocyte migration, transplantation, graft rejection

## Abstract

Immunological memory is a key feature of adaptive immunity. It provides the organism with long-lived and robust protection against infection. In organ transplantation, memory T cells pose a significant threat by causing allograft rejection that is generally resistant to immunosuppressive therapy. Therefore, a more thorough understanding of memory T cell biology is needed to improve the survival of transplanted organs without compromising the host’s ability to fight infections. This review will focus on the mechanisms by which memory T cells migrate to the site where their target antigen is present, with particular emphasis on their migration to transplanted organs. First, we will define the known subsets of memory T cells (central, effector, and tissue resident) and their circulation patterns. Second, we will review the cellular and molecular mechanisms by which memory T cells migrate to inflamed and non-inflamed tissues and highlight the emerging paradigm of antigen-driven, trans-endothelial migration. Third, we will discuss the relevance of this knowledge to organ transplantation and the prevention or treatment of allograft rejection.

## Introduction

Immunity is the balanced state of having adequate protection against infection, while maintaining adequate tolerance to avoid excessive inflammation, allergy, and autoimmune diseases. In most vertebrates including humans, the immune system is composed of two principal components: innate and adaptive. Relatively non-specific innate immune responses proceed prior to the development of more specific adaptive immunity – thus, providing immediate protection against invading microbes. However, a more long-lasting and robust protective strategy is needed. Unlike innate immunity, adaptive immunity generates immunological memory after an initial response to a pathogen or non-self molecules from genetically unrelated individuals (alloantigens). Immunological memory provides the organism with a faster and stronger protective response upon reencountering the same pathogen. In the organ transplantation setting, however, allo-reactive memory T cells pose a significant threat by mediating accelerated, “second-set” rejection and its presence has been associated with increased propensity for early rejection (1). Furthermore, when compared to naive T cells, memory cells are more resistant to immunosuppressive therapies as their reactivation and expansion require less costimulation and are independent of secondary lymphoid organs (SLOs); therefore, they are more efficient at driving effector functions that lead to graft injury (2, 3). A more thorough understanding of memory T cell biology should provide new insights into graft rejection. In this review, we will focus on connecting the well-established memory T cell chemokine-driven migration paradigm with new findings in this field, emphasizing the role of cognate antigens present in target tissues. We will also discuss the necessity of and rationale for the two complementary mechanisms that underlie memory T cell migration.

## Memory T cell Subsets and Their Circulation Patterns

The heterogeneity of the memory T cell population was first recognized approximately two decades ago. As initially stated, human peripheral blood memory T cells are comprised of two subsets with distinct homing potentials and effector functions. CCR7^−^ memory T cells express low levels of the CD62L molecule, migrate to inflamed non-lymphoid tissues, and display immediate effector function such as IFN-γ production – thus, they earned themselves the moniker effector memory (T_EM_). CCR7^+^ memory T cells, on the other hand, express high levels of CD62L, which along with CCR7 is a lymph-node homing receptor, and were named central memory (T_CM_). T_CM_ are mainly found in lymphoid tissues and lack immediate effector function; however, they produce IL-2 and proliferate extensively upon re-stimulation, whereas T_EM_ display less proliferative capacity (4). Subsequently, the central and effector memory T cell subset concept was shown to have parallels in mice (5, 6). A more recent study indicated that fundamental mechanisms of T cell memory in humans and mice share much in common (7). Thus, knowledge acquired in mouse and human memory studies appears to be interchangeable. Significant findings in mouse memory studies, therefore, have the potential of rapid translation to non-human primates and humans (8).

More recently, in-depth studies on local immunity identified a new memory T cell subset, tissue-resident memory (T_RM_), which resides in peripheral non-lymphoid tissues long after the initial infection has cleared. T_RM_ provide frontline local protective immunity when the same pathogen is reencountered at the entry site. Although they express low levels of CCR7 and CD62L as do T_EM_, T_RM_ express high levels of local non-lymphoid tissue-homing molecules such as CD103 and CD69 (9, 10). T_RM_ do not routinely recirculate and mix with memory subsets in other tissues (10), which is not surprising, given the fact T_RM_ were initially missed in studies investigating the peripheral blood or secondary lymph organs (SLOs).

In summary, the three memory subsets (T_EM_, T_CM_, and T_RM_) have distinct circulation patterns as a result of their distinct surface chemokine receptor and adhesion molecule expression. T_CM_ mainly circulate in lymphoid tissues (lymph nodes, spleen, and bone marrow) and blood; T_EM_ can circulate between lymphoid tissues and non-lymphoid tissues during steady state conditions (although they are largely excluded from lymph nodes) and can skew their preference under certain contexts, which will be discussed in detail below; T_RM_ only reside in peripheral non-lymphoid tissues and do not circulate as their counterparts do (8). In this review, we will be mainly discussing T_EM_ migration. Although there are some studies indicating that T_CM_ proliferation and differentiation under both steady and challenged states can give rise to T_EM_ and maintain the labile T_EM_ repertoire (11, 12), they will not be discussed here.

## Cellular Mechanisms Underlying Memory T cell Migration

Memory T cells within the circulatory system specifically T_EM_ are constantly on the move for the purpose of immune surveillance. Their recruitment to peripheral tissues requires adhesion to and transmigration across blood-vessel walls. This adhesion-migration cascade consists of four continuous steps: slow rolling, adhesion strengthening (firm adhesion), and intraluminal crawling followed by paracellular and transcellular migration (13). The underlying mechanisms of cascade activation have been intensively investigated. Slow rolling along the endothelium under blood flow condition is mediated by L-selectin, P-selectin, and E-selectin, which mainly interact with P-selectin glycoprotein ligand 1 (PSGL-1). L-selectin is expressed by leukocytes, while P-selectin and E-selectin are mainly expressed by inflamed endothelial cells (ECs) (13). It has long been recognized that lymphocyte arrest during rolling is rapidly triggered by chemokines or other chemoattractants and is mediated by the binding of lymphocyte integrins to immunoglobulin superfamily members, such as ICAM1 and VCAM1, expressed by ECs. This chemokine-driven paradigm is dependent on signaling in T cells via the G-protein Gαi, which is coupled to chemokine receptors (14, 15). This process, the molecular details of which will be explained in the next section, is largely enhanced where local inflammation is established as chemokines and other chemoattractants are locally upregulated and displayed on ECs by binding to glycosaminoglycans (GAGs). It has been shown that T cell homing to different peripheral tissues express distinct sets of homing molecules, corresponding to the different chemokines and adhesion molecules displayed on the endothelium in each specific inflamed tissue. Skin-homing T cells mainly express the chemokine receptors CCR4 and CCR10, which bind to their ligands CCL17 and CCL27 present in the dermis and epidermis, respectively (16–18). As migration of CD4 and CD8 T cells to the skin follows anatomic demarcations, unique expression of chemokine receptors on CD4 and CD8 T cells may reflect their preferential skin-migratory capacity, with CCR10-expressing CD8 T cells entering the epidermis and CCR4-expressing CD4 T cells accessing the dermis (17–19). Distinctively, gut-homing T cells express CCR9 and are attracted by the CCR9 ligand CCL25 expressed by intestinal epithelial cells (20).

Initial studies have indicated that the increased recruitment of T lymphocytes during inflammation is not necessarily antigen-specific as both antigen-specific and bystander memory T cells accumulate in inflamed peripheral tissues. However, the antigen-specific T cell population is preferentially retained and activated, while the bystander T cell number gradually declines to a background level and they retain a phenotypically inactive state (21, 22). In a recent study, using a B6-OVA to B6 transplant model where one single antigenic difference between the donor and recipient exists, it has been shown that cognate antigen presence is necessary for driving antigen-specific memory T cell migration into the peripheral tissue, irrespective of whether acute inflammation is present or not, followed by the migration of bystander T cells (23). The entry of the former was not dependent on Gαi, as it was not inhibited by pertussis toxin, while the entry of the latter was dependent on Gαi-coupled chemokine receptors. Bystanders would migrate into the tissue only if antigen-specific T cells were already present in the tissue where they presumably cause local inflammation and induce chemokine production. This emerging Ag-driven memory T cell migration paradigm is also true in an autoimmune context where inflammation is minimal at the initial stages of T cell accumulation in the pancreatic islets. Until then, it had been widely believed that islet infiltrating T cells in diabetic mice are composed of both islet antigen-specific as well as bystander T cells, and the heterogeneous recruitment was driven by inflammatory chemokines (24). However, in a study where TCR retrogenic mice were used, it was shown that only islet antigen-specific T cells accumulate in the pancreatic islets while bystander T cells do not even after the accumulation of antigen-specific T cells. In addition, islet Ag-specific T cell entry did not necessarily cause diabetes since only diabetogenic T cell infiltration caused diabetes while non-diabetogenic autoantigen-specific T cells did not. Therefore, the authors of this study concluded that islet antigen specificity mediated a cell-autonomous and tightly regulated event and was the key for pancreatic islet accumulation (25). In later studies, Calderon et al. demonstrated that CD4 TCR-transgenic T cells that are specific to islet antigens enter pancreatic islets in a manner that is not dependent on Gαi-coupled chemokine receptor signaling (26). The entry of bystander T cells that are not specific to islet antigens, however, was driven by the subsequent wave of chemokines induced by IFNg secreted by the islet antigen-specific T cells (27). Moreover, it has been shown that both dendritic cells (DCs) and ECs can present cognate antigen to memory T cells, conferring the T cell transmigration driving force in the contexts of both transplantation and autoimmunity (23, 26).

To summarize, Gαi-dependent chemokine receptor signaling and cognate antigen-engaged TCR signaling are two complementary pathways underlying memory T cell migration, as they both trigger downstream integrin activation, e.g., LFA-1 and VLA-4 (28, 29). Integrin conformation change and clustering are essential for memory T cell migration, and LFA-1 or VLA-4 blockade results in significant reduction in T cell migration (30). The chemokine receptor and TCR-driven migration paradigms may be differentially required at different stages of infiltration process, as antigen recognition seems to be key for early T cell infiltration, while chemokine signaling comes into play at a later stage (23, 25, 31). In the next section, we will discuss the intracellular events that underlie these two pathways that trigger integrin activation in T cells.

## Molecular Mechanisms Underlying Memory T Cell Migration

Understanding the molecular mechanisms responsible for T cell migration is important for identifying targets to block T cell entry into non-lymphoid tissues in pathologic states such as autoimmunity and graft rejection. Careful dissection of the intracellular signaling pathways triggered by chemokine receptors versus antigen receptors would also pave the way for specifically blocking antigen-dependent migration of T cells while sparing chemokine-driven migration. Since the latter appears to be the main pathway of T cell migration to sites of microbial infection, selectively blocking the former could help treat patients with autoimmunity or organ transplant rejections without increasing the risk of infection. Therefore, in this section, we will discuss in detail how signaling molecules triggered by chemokine receptors differ from those triggered by antigen receptors.

**Figure 1:**
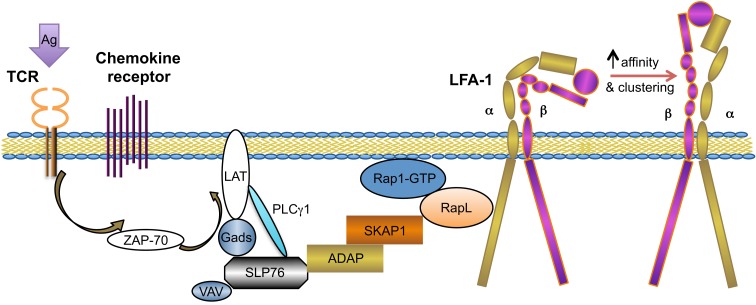
**TCR-mediated integrin activation inside-out signaling**. This figure outlines the key mediators in the TCR signaling-mediated integrin (LFA-1) activation pathway. ADAP and SKAP1 are two adaptor proteins involved in this pathway, functioning by synergistically recruiting RapL–Rap1–GTP complex to the integrin cytosolic tail and leading to integrin confirmation changes and membrane clustering [modified from Ref. (34)].

As noted previously, integrin activation is essential for T cell arrest and firm adhesion on blood vessel walls, which are critical steps that trigger memory T cell transmigration across the endothelium and into peripheral tissues. There are two well-known modalities of integrin activation: conformational changes leading to increased affinity of individual integrin molecule and lateral mobility (clustering) of the integrin molecules contributing to an overall enhanced cell avidity (Figure 1) (32). Structural studies of LFA-1, a common integrin involved in cell adhesion and migration, indicated that integrins dynamically equilibrate in three distinct conformational states, which are designated as folded, extended-closed, and extended-open conformations. Both folded and extended-closed integrins display relatively low affinity, while extended-open integrin affinity is 10^3^–10^4^-fold higher. These integrin conformational changes are consequences of inside-out signaling: signals that are first triggered by a receptor on the cell surface (outside-in) but then signal from inside the cell (inside-out) to activate integrin molecules (33).

There are mainly two types of receptors that trigger the signals involved in integrin conformational changes, namely G protein-coupled chemokine receptors that bind chemokines such as CCL19, CCL21, or CXCL12, and lymphocyte receptors for antigen (TCRs and BCRs) with some influence from costimulatory receptors (35, 36). In the chemokine-signaling cascade, dozens of signaling proteins have been implicated in regulating distinct aspects of integrin activation (conformation change, structural stabilization, etc.). Most notably, both rap and rho have been validated in transducing signals in the chemokine-mediated integrin activation pathway. Rap and rho are small GTP binding proteins belonging to the ras superfamily. Distinctly, rap and rho each regulate different signal modules, leading to specific control of integrin affinity (37). The rap isoform Rap1A is downstream of G protein-coupled receptor (GPCR)-induced rapid intracellular calcium influx and activation of phospholipase C (PLC), and its activation is directly regulated by RAPGEF2, whose activity is controlled by PLC-triggered further up-regulation of intracellular calcium and increased intracellular diacylglycerol (DAG) (38). RapL is a binding protein of Rap1A, and its overexpression enhances LFA-1 clustering and adhesion (39). The mechanism via rho-module is less well-defined, although a role of rho isoform RhoA in LFA-1 activation has been identified many years before (40). Protein kinase C (PKC) isozyme, ζPKC is probably one of the candidates, as it directly interacts with RhoA and has been shown to be involved in LFA-1 clustering (41, 42).

As the TCR-driven T cell migration paradigm has been gradually emphasized over the past decade, here, we will discuss TCR-mediated integrin activation in detail (Figure 1). TCR engagement-mediated integrin activation facilitates T cell adhesion to MHC-bearing antigen presenting cells (APCs), stabilizing the T-APC conjugates. The interfaces are referred to as immunological synapses (43). The immunological synapse is characterized by the formation of a supramolecular adhesion complex (SMAC), which is comprised of a central concentric ring (c-SMAC) of TCR-CD3 and costimulatory receptors, and a peripheral concentric ring (p-SMAC) of LFA-1 and the cytoskeletal protein talin. The stabilization of this dynamic structure is correlated with full T cell activation and effector T cell function (44, 45). Integrin activation initiated by TCR signaling occurs within minutes following T-cell stimulation and the underlying intracellular mechanisms have been extensively investigated. The initiation of integrin activation signaling cascades by TCR-proximal signaling events are mediated by the linker for activation of T cells (LAT) adapter protein, as LAT-deficient T cells show impaired TCR signaling and arrested T cell development. LAT is a substrate of the tyrosine kinase activated following TCR engagement (46). LAT can bind or recruit multiple signaling proteins, such as tyrosine kinase Itk and PLC-γ1, and both these two LAT-associate proteins play critical roles in TCR-mediated integrin activation, possibly by facilitating optimal PLC-γ1 phosphorylation and activation (29). PLC-γ1 is shown to be specifically involved in the TCR inside-out signaling to integrin, rather than the chemokine signaling to integrin (47). Although the indispensable role of PLC-γ1 in TCR-integrin signaling has been established, the mechanism how PLC-γ1 regulate integrin activation remains elusive (48). Additionally, SLP-76 is recruited to the LAT complex upon TCR stimulation via the adapter protein GADS (49). SLP-76 is an adapter protein not only regulating PLC-γ1 activation but also recruiting another important adapter protein, adhesion and degranulation promoting adapter protein (ADAP), involved in the TCR-integrin signaling cascade (50, 51). Adapter ADAP is a hematopoietic cell-specific protein, mainly expressed in mast cells and T cells. It has recently been established that ADAP regulates integrin-mediated adhesion in T cells (52). Genetic deficiency of ADAP results in impaired proliferative responses and decreased effector functions in T cells, thus leading to prolonged allograft survival (53). In T cells, ADAP is constitutively associated with another adapter partner, namely, SKAP55/SKAP1 (Src kinase-associated adapter protein of 55 kDa), which is prominently expressed in T cells. The interaction involves the SH3 domain of SKAP1 and the proline-rich region in ADAP (54, 55). The ADAP/SKAP1 signaling module regulates TCR-mediated integrin activation through plasma membrane recruitment of activated Rap1 (56). Importantly, SKAP1 has been shown to have an indispensable role in LFA-1 clustering on T cells (57). RapL (regulator of cell adhesion and polarization enriched in lymphoid tissues), one of the binding partners of Rap1, is indicated to bind to the N-terminus of SKAP1 via its C-terminal SARAH domain (58). This finding is indicative of the underlying mechanism by which the ADAP/SKAP1 signaling module recruits RapL–Rap1 complex to the membrane. Furthermore, RIAM (Rap1–GTP-interacting adapter molecule) is another important adapter protein, which links Rap1 and talin, which is the most common cytoplasmic integrin-associated actin-binging protein. Talin is proposed to be the “final common step” in integrin activation, both in chemokine-mediated and TCR-mediated integrin activation (29).

## Relevance to Organ Transplantation

In transplantation, allo-reactive memory T cells contribute to both acute and chronic rejection; therefore, they pose a significant barrier to graft acceptance (59). Allo-reactive memory T cells are either pre-existing or *de novo* generated (60). Pre-exposure to allo-antigen (such as pregnancy, transfusion, and previous organ transplants) is one reason but not the only reason for pre-existence of allo-reactive memory T cells (61). Many studies have shown that memory T cells widely display cross-reactivity, which is also known as heterologous immunity. A proportion of memory T cells generated against encountered pathogens in one’s life can actually show cross-reactivity to alloantigens such as the HLA (62, 63). Not only are memory T cells resistant to conventional immunosuppressive therapies such as costimulation blockade and chimerism based therapies, but also they are relatively resistant to regulation by CD4+ CD25+ FoxP3+ regulatory T cells (Treg), which makes them foe of tolerance induction and long-term graft survival (64).

Pre-existing donor-reactive memory T cells infiltrate allograft rapidly post transplantation and gain effector functions bypassing the need for SLOs (3). Since memory T cell graft infiltration is a prerequisite for memory T cell-mediated graft rejection, blockade of their migration to the graft should be a promising therapy in transplantation (65). As previously mentioned, integrin is indispensably involved in T cell peripheral migration process. However, monoclonal antibodies that target LFA-1, for example, block memory and effector T cell migration indiscriminately and their combination with a standard immunosuppressive regimen increases the chance of developing post transplant EBV-associated lymphoproliferative disease, even though they significantly attenuate T cell trafficking to graft (66). Monoclonal antibodies that target VLA-4 are also associated with reactivation of fatal infections. Thus, it is necessary to seek other strategies to inhibit memory T cell migration without increasing the risk of infection. One such strategy would be to target the inside-out signaling pathway downstream of the TCR but not downstream of chemokine receptors – thus, inhibiting antigen-driven but not chemokine-driven T cell migration. SKAP1, for example, would be a candidate target molecule to stop the antigen-driven migration of allo-reactive T cells. Additional animal studies should help test this possibility with the promise of specifically inhibiting alloreactive memory while sparing “good memories” against pathogens.

## Conflict of Interest Statement

The authors declare that the research was conducted in the absence of any commercial or financial relationships that could be construed as a potential conflict of interest.
